# Full-root aortic valve replacement with stentless xenograft achieves superior regression of left ventricular hypertrophy compared to pericardial stented aortic valves

**DOI:** 10.1186/s13019-015-0219-8

**Published:** 2015-02-03

**Authors:** Reza Tavakoli, Christoph auf der Maur, Xavier Mueller, Reinhard Schläpfer, Peiman Jamshidi, François Daubeuf, Nelly Frossard

**Affiliations:** 1 Department of Cardiac Surgery, Canton Hospital Lucerne, Lucerne, Switzerland; 2 Institute of Veterinary Pysiology Vetsuisse Faculty and Zurich Center for Integrative Human Physiology, University of Zurich, Zurich, Switzerland; 3 Department of Cardiology, Canton Hospital Lucerne, Lucerne, Switzerland; 4 Laboratoire d’Innovation Thérapeutique, Unité Mixte de Recherche 7200, Centre National de la Recherche Scientifique-Université de Strasbourg, Faculté de Pharmacie, Illkirch, Strasbourg, F-67400 France

**Keywords:** Full-root stentless aortic xenograft, Left ventricular mass index

## Abstract

**Background:**

Full-root aortic valve replacement with stentless xenografts has potentially superior hemodynamic performance compared to stented valves. However, a number of cardiac surgeons are reluctant to transform a classical stented aortic valve replacement into a technically more demanding full-root stentless aortic valve replacement. Here we describe our technique of full-root stentless aortic xenograft implantation and compare the early clinical and midterm hemodynamic outcomes to those after aortic valve replacement with stented valves.

**Methods:**

We retrospectively compared the pre-operative characteristics of 180 consecutive patients who underwent full-root replacement with stentless aortic xenografts with those of 80 patients undergoing aortic valve replacement with stented valves. In subgroups presenting with aortic stenosis, we further analyzed the intra-operative data, early postoperative outcomes and mid-term regression of left ventricular mass index.

**Results:**

Patients in the stentless group were younger (62.6 ± 13 *vs*. 70.3 ± 11.8 years, p < 0.0001) but had a higher Euroscore (9.14 ± 3.39 *vs.*6.83 ± 2.54, p < 0.0001) than those in the stented group. In the subgroups operated for aortic stenosis, the ischemic (84.3 ± 9.8 *vs.* 62.3 ± 9.4 min, p < 0.0001) and operative times (246.3 ± 53.6 *vs.* 191.7 ± 53.2 min, p < 0.0001) were longer for stentless versus stented valve implantation. Nevertheless, early mortality (0% *vs.* 3%, p < 0.25), re-exploration for bleeding (0% *vs.* 3%, p < 0.25) and stroke (1.8% *vs.* 3%, p < 0.77) did not differ between stentless and stented groups. One year after the operation, the mean transvalvular gradient was lower in the stentless *versus* stented group (5.8 ± 2.9 *vs.* 13.9 ± 5.3 mmHg, p < 0.0001), associated with a significant regression of the left ventricular mass index in the stentless (p < 0.0001) but not in the stented group (p = 0.2).

**Conclusion:**

Our data support that full-root stentless aortic valve replacement can be performed without adversely affecting the early morbidity or mortality in patients operated on for aortic valve stenosis provided that the coronary ostia are not heavily calcified. The additional time necessary for the full-root stentless compared to the classical stented aortic valve replacement is therefore not detrimental to the early clinical outcomes and is largely rewarded in patients with aortic stenosis by lower transvalvular gradients at mid-term and a better regression of their left ventricular mass index.

## Background

An increasing number of patients are requiring aortic valve replacement, in part because of the aging population. Biological aortic valve replacement is recommended for patients older than 65 years [1]. Stented aortic bioprostheses have been used for decades with good long-term results [2]. Yet, the ideal biological aortic valve substitute remains a subject of debate. Aortic homografts have been implanted by experienced surgeons with similarly excellent long-term results [3]. Because of the limited availability and the reported late degeneration of the aortic homografts [4], stentless aortic xenografts have been developed to provide with similar hemodynamic profile and equal or better durability. Stentless aortic xenografts can be implanted as a subcoronary, as a root inclusion or as a full-root aortic valve replacement [5]. Full-root replacement technique has potentially superior hemodynamic performance compared to subcoronary implantation of stented or stentless aortic valve substitutes, since i) it offers the possibility to implant a 3–4 mm larger valve in a given patient, thus allowing significant reduction in transvalvular gradients resulting in a better regression of left ventricular hypertrophy, ii) the preservation of the sinuses of the porcine aortic root maintains an optimal coronary perfusion, hence promoting further improvement in patient outcomes, iii) these performances apply equally to younger patients.

Nevertheless, a number of cardiac surgeons are reluctant to transform a classical aortic valve replacement with stented biological valve into a technically more demanding procedure represented by full-root replacement with stentless aortic xenografts. Moreover, there are concerns regarding the increased potential for bleeding and for possible distortion of coronary ostia anastomoses. Here, we report our experience of full-root replacement with stentless aortic xenografts with emphasis on the surgical technique. In the subgroups of patients with aortic stenosis, the early postoperative outcomes, transvalvular gradients and extent of regression of the left ventricular mass index at one year post-operatively in these patients are further compared retrospectively to those of patients who underwent stented aortic valve implantation during the same period by the same surgeon.

## Methods/Design

### Patient population

From January 2005 to December 2013, one hundred and eighty patients underwent full-root replacement with stentless porcine aortic xenografts by a single surgeon (RT) according to the technique described here. Stentless aortic xenografts used were either Edwards Prima Plus (Edwards Lifescience, Irvine, CA, USA) or Medtronic Freestyle (Medtronic Inc., Minneapolis, MN, USA). Patients’ characteristics were compared retrospectively to those of 80 patients who underwent aortic valve replacement by sternotomy with stented bovine pericardial aortic valves (Carpentier-Edwards Perimount, Magna, Magna ease, Edwards Lifescience, Irvine, CA, USA) during the same period by the same surgeon (Table 1). Further, in the subgroups of patients with aortic stenosis, the peri-operative data and hemodynamic performance at one year after the operation determined by echocardiography were compared between the stentless and stented cohorts. Echocardiographic data collection was closed in December 2013. Therefore, echocardiographic data of the patients operated in 2013 were not included in the one-year comparison.

**Table 1:** **Patient characteristics for the whole study population**

	Full root stentless xenografts	Stented valves	p value
	n = 180	n = 80	
Age, years	62.6 ± 13	70.3 ± 11.8	0.0001
Female gender	49(27%)	29(36%)	0.1437
Ejection fraction	53 ± 11	55 ± 8	0.1043
Redo surgery	41(23%)	6(7.5%)	0.0032
Pathology			
Aortic stenosis	75(41%)	64(80%)	0.0001
Aortic regurgitation	44(24%)	14(17.5%)	0.2158
Native endocarditis (6AS)	30(16%)	0	0.0001
Prosthetic endocarditis	14(7%)	0	0.0001
Prosthetic dysfunction	12(6%)	0	0.0001
Aortic dissection	11(6%)	2(2.5%)	0.2193
Isolated aortic valve procedures	85(47%)	36(45%)	0.7415
Concomitant procedures	95(53%)	44(55%)	0.7414
Priority			
Elective	108(60%)	78(97.5%)	0.0001
Urgent	50(27%)	0	0.0001
Emergency	22(13%)	2(2.5%)	0.0125
Euroscore	9.14 ± 3.39	6.83 ± 2.54	0.0001

### Surgical technique

All patients underwent median sternotomy. After heparinization, cardiopulmonary bypass was established between the distal ascending aorta or the right subclavian artery and the right atrium or percutaneously the femoral vein. The cardioplegic route was secured anterogradely (direct coronary ostia when indicated) and retrogradely through the coronary sinus. The ascending aorta was cross-clamped and cold blood cardioplegia delivered and repeated during the procedure.

Our practical approach to select the valve substitute for a given patient was guided by the intention to achieve a projected indexed effective orifice area (IEOA) ≥ 0.85 cm^2^/m^2^ based on the EOA provided by the manufacturer to avoid as far as possible patient-prosthesis mismatch (PPM). The final decision at the moment of implantation was derived from the perceived balance between the potentially increased peri-operative risk of a more demanding operation represented by full-root replacement, and the potential advantages for the patient in terms of quality of life and long-term survival.

### Stentless aortic xenograft

**Figure 1:**
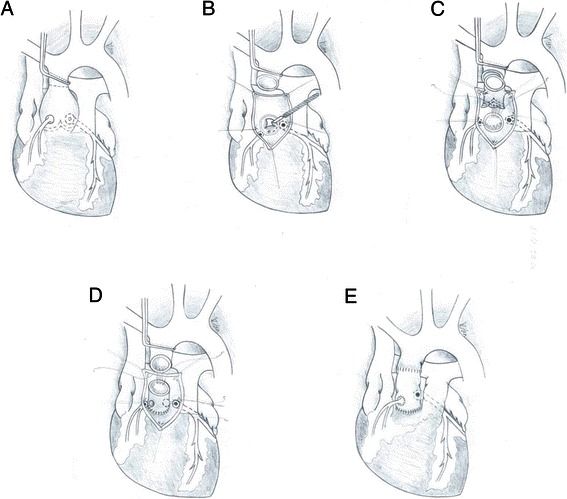
**Surgical technique of full-root aortic xenograft implantation.** The aorta is cross-clamped above the sino-tubular junction and transected at its level to inspect the aortic valve and root **(a)**. The aortic valve is removed and the coronary ostia are dissected free from the aortic sinuses with a generous patch **(b)**. The aortic stentless xenograft is implanted onto the surgical aortic annulus with 6 Prolene 4.0 running sutures beginning under the left coronary ostium in a clockwise manner **(c)**. The coronary ostia are reimplanted into the stentless xenograft in a manner to avoid tension, torsion or kinking of the patient’s proximal coronary arteries **(d)**. The implantation of the xenograft is completed by end-to-end anastomosis between the stentless xenograft and the mobilized distal aorta with a running Prolene 5.0 suture **(e)**.

The ascending aorta was transversally transected 1 cm-above the right coronary ostium (Figure 1a). After inspection, the aortic valve was removed and the aortic annulus prepared free as needed. The coronary ostia were dissected free from the aortic wall with sufficient surrounding patch, and mobilized as far as possible (Figure 1b). The aortic stentless xenograft was implanted onto the surgical aortic annulus with 6 Prolene 4.0 running sutures (Figure 1c). The first suture was placed at the deepest point of the left coronary sinus so that the left coronary button hole of the xenograft exactly faced the left coronary ostium of the patient. The suture was then run up to the commissure between the left and right coronary sinuses and continued by a second suture in a clockwise manner to the middle of the right coronary sinus. Thus, each sinus of the xenograft was secured to the aortic annulus with two running sutures (Figure 1c). As the height and the angle between the native porcine coronary ostia are different from those in the normal human anatomy, new ostia were fashioned in the xenograft in a manner to avoid tension, torsion or kinking of the patient’s proximal coronary arteries. The angle between the coronary arteries is more acute in porcine than in human aortic root. To correct this mismatch, we cut the left button hole in the xenograft rather closer to the commissure between the left and non-coronary cusp and the right button hole in the xenograft rather closer to the commissure between the right and non-coronary cusp. The left button hole in the xenograft was placed close to the aortic annulus, and the right button hole close to the free edge of the xenograft. The coronary ostia of the patient were then attached to the corresponding button hole of the xenograft with a 6.0 Prolene running suture (Figure 1d). The implantation of the xenograft was completed by end-to-end anastomosis between the xenograft and the mobilized distal aorta with a running Prolene 5.0 suture (Figure 1e). Heavily calcified coronary ostia represented technical limitations to this technique.

#### Stented pericardial aortic valve

Stented valves were implanted in the subcoronary position with pledgeted 2/0 Ethibond U-sutures (Ethicon, Johnson&Johnson, Sollentuna, Sweden).

### Echocardiographic data

Two-dimensional transthoracic echocardiography was used to measure transvalvular gradients and left ventricular mass index during the planned one-year post-operative follow-up. Standard apical, long-axis and short-axis views, together with Doppler flow measurements were performed.

### Statistical analysis

Continuous variables were expressed as means ± SD and compared between groups using the non-parametric Mann Whitney test. Categorical variables were expressed as percentages and compared between groups using the Chi-square test. Pre- *versus* post-operative data (LVMI) were compared using the Wilcoxon matched-pair signed rank test. Statistical analysis was performed using Prism software (GraphPad, La Jolla, CA, USA), with statistical significance set at p < 0.05.

## Results

### Patients’ characteristics

In the whole study population, patients in the stentless group had a higher Euroscore although they were younger than those in the stented group [6,7]. This was due to the fact that there were more redo and/or urgent operations in the former group. Aortic stenosis was the underlying pathology in 41% of the patients in the stentless *versus* 80% in the stented group. Isolated aortic valve replacement was performed in 47% of the patients in the stentless and in 45% of the patients in the stented group (Table 1).

In the stentless group 75 patients had aortic stenosis. Of these 75 patients, 68 were operated before January 2013 and could be studied by echocardiography at one year. There were no death peri-operative and until one year at the time of echocardiography among these patients. For these 68 patients, the follow-up was 100% complete.

In the stented group 64 patients had aortic stenosis. Of these 64 patients, 46 were operated before January 2013 and could have been available for echocardiography at one year. Among these 46 patients, 36 patients underwent isolated stented aortic valve replacement with one peri-operative death. There was no other death among the remaining 45 patients who were studied by echocardiography at one year. For these 45 patients, the follow-up was 100% complete.

Subgroups of patients with aortic stenosis with or without coronary artery disease were further analyzed for the peri-operative outcomes and mid-term hemodynamic performances. In contrast to the whole study population, there was no difference in the risk profile, reflected by Euroscore, in these subgroups of patients between the stentless and stented cohorts (Table 2).

**Table 2:** **Patient characteristics for the subgroups of patients with aortic stenosis with or without associated coronary artery disease operated before January 2013**

	Full root stentless xenografts	Stented valves	p value
	n = 68	n = 46	
Age, years	59.9 ± 11.6	76.1 ± 5.7	0.0001
Female gender	19(28%)	25(54%)	0.0045
Ejection fraction	56.7 ± 8	56.5 ± 7.6	0.8936
Redo surgery	15(22%)	4(9%)	0.0603
Isolated aortic valve procedures	55(81%)	36(78%)	0.7322
Elective	64(94%)	46(100%)	0.0940
Euroscore	8 ± 2	7.3 ± 1.6	0.1113
Arterial hypertension	24(35%)	18(39%)	0.2011

Of the 68 stentless patients with aortic stenosis studied at one year, 55 had isolated full-root aortic valve replacement. Operative and early post-operative data of these 55 patients were compared to those of 36 patients who underwent isolated stented aortic valve replacement (Table 3). In these patients, the ischemic time for isolated full-root stentless xenograft implantation was significantly longer than that for stented aortic valve replacement (84.3 ± 9.8 *versus* 62.3 ± 9.4 minutes, p < 0.0001) (Table 3). Accordingly, the cardiopulmonary bypass and operative times were respectively 36 and 55 minutes longer for the former group. Despite the more demanding procedure for full-root stentless xenograft implantation, peri-operative complications and early death rates were comparable to those for stented aortic valve replacement (Table 3).

**Table 3:** **Operative and early post-operative data for isolated procedures in patients with aortic stenosis operated before January 2013**

	Full-root stentless xenografts	Stented pericardial valves	p
	N = 55	N = 36	
Age, years	62.8 ± 10.9	71.6 ± 10.1	0.0004
Female gender	10(18%)	15(42%)	0.0141
Ejection fraction	51 ± 13	57 ± 8	0.0153
Redo surgery	4(7%)	4(11%)	0.5272
Cross-clamp time (min)	84.3 ± 9.8	62.3 ± 9.4	0.0001
CPB time (min)	137 ± 34.5	101 ± 27.2	0.0001
OP time (min)	246.3 ± 53.6	191.7 ± 53.2	0.0001
Re-exploration for bleeding	0	1(3%)	0.2249
Pace maker	1(1.8%)	0	0.4315
Stroke	1(1.8%)	1(3%)	0.7736
Sternal infection	0	0	ns
Early mortality	0	1(3%)	0.2249

**Figure 2:**
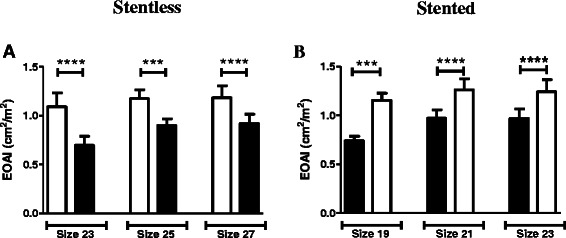
**Projected*versus*calculated IEOA in patients with aortic stenosis operated before January 2013.A)** The projected IEOA for patients in the stentless group (white bars) was compared to the calculated IEOA if they had received a stented pericardial valve 3–4 mm smaller (black bars). **B)** The projected IEOA for patients in the pericardial stented group (black bars) was compared to the calculated IEAO if they had received a full-root stentless aortic valve 3–4 mm larger (white bars). Blocks are means and bars are SD values. ***indicates p < 0.001 and ****p < 0.0001.

For patients with aortic stenosis, the projected indexed effective orifice areas (IEOA) based on the effective orifice areas for different sizes of stentless and stented pericardial aortic valves supplied by the manufacturers are shown in Figure 2 and Table 4. The projected IEOA for patients in the stentless group was significantly higher than the calculated IEOA if they had received a stented pericardial valve 3–4 mm smaller (Figure 2A). Conversely, the projected IEOA for patients in the pericardial stented group was significantly lower than the calculated IEAO if they had received a full-root stentless aortic valve 3–4 mm larger (Figure 2B).

**Table 4:** **Hemodynamic data for the subgroups of patients with aortic stenosis with or without associated coronary artery disease operated before January 2013**

	Full root stentless xenografts	Stented valves
	n = 68	n = 45
1-year mean gradient (mmHg)	5.7 ± 2.9	13.9 ± 5.3
1-year maximum gradient (mmHg)	10.7 ± 5.7	23.9 ± 9.6
Projected Indexed EOA (cm^2^/m^2^)	1.16 ± 0.11	0.92 ± 0.12
Preoperative LVMI (g/m2)	156 ± 16	133 ± 37
1-year LVMI (g/m2)	103 ± 16	124 ± 33

**Figure 3:**
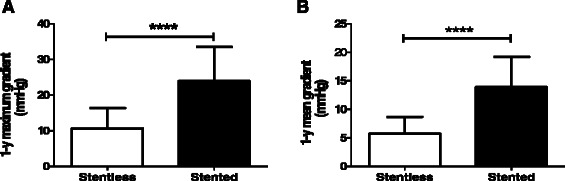
**Post-operative transvalvular gradients in patients with aortic stenosis operated before January 2013.A)** Maximum transvalvular gradients at one year after the operation for patients in the stentless compared in the pericardial stented group. **B)** Mean transvalvular gradients at one year after the operation for patients in the stentless compared in the pericardial stented group. Blocks are means and bars are SD values. ****indicates p < 0.0001.

**Figure 4:**
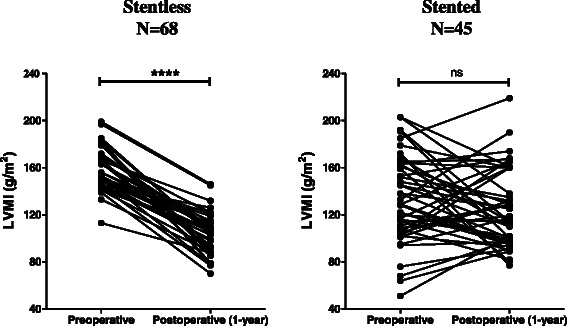
**Pre- and post-operative LVMI in patients with aortic stenosis operated before January 2013.** The left ventricular mass index at one year after the operation was compared to the pre-operative value in the stented group (left panel) and in the stentless group (right panel) using the non- parametric Wilcoxon matched-pair signed rank test. Ns, non-significant, and ****indicates p < 0.0001.

In patients with aortic stenosis, the maximum and mean transvalvular gradients at one year after the operation were significantly lower for the stentless aortic xenografts as compared to the pericardial stented aortic valves (Figure 3A and B). In correlation with this observation, compared to the pre-operative finding, the post-operative LVMI at one year was significantly reduced in patients who received full-root aortic xenografts. In contrast, in patients who received pericardial stented aortic valves, the reduction in the post-operative LVMI at one year compared to the pre-operative finding did not reach statistical significance (Figure 4).

## Discussion

This study reports a detailed description of the surgical technique of full-root aortic valve replacement using stentless aortic xenografts in 180 consecutive patients. In a subgroup of patients with aortic stenosis, we compared the early clinical outcomes, mid-term transvalvular gradients and degree of LVMI regression to those of patients who underwent aortic valve replacement with stented aortic valves.

In our technique, the proximal (inflow) anastomosis for full-root implantation of the stentless aortic xenograft is performed using a total of 6 Prolene 4.0 running sutures (2 running sutures per sinus of Valsalva) without any reinforcement with pericardial or Teflon strips, as described by other authors [8-10]. Compared to multiple simple interrupted sutures [10,11] and a single running suture [8,9], our use of 6 running sutures allows for a more controlled and meticulous implantation of the stentless aortic xenograft at the proximal anastomosis site. In our whole series of 180 patients, no resternotomy was necessary for bleeding. Our technique of reimplantation of the coronary arteries allowed us to avoid any tension on and malalignment of the coronary arteries in patients. Other authors rotate the stentless xenograft in a clockwise manner in order to direct the right coronary ostium of the graft to the non-coronary sinus of the patient [9]. This rotation necessitates oversewing of the right coronary ostium of the graft. Our technique of coronary reimplantation also used by the majority of authors [8,10,11] simplifies this part of the procedure.

This report represents one of the largest series of full-root replacement using stentless xenografts performed by a single surgeon [9,11-13]. Involvement of only one surgeon eliminates one of the biases of the studies comparing stented aortic valves to stentless aortic xenografts implanted as a full-root or in the sub-coronary position, including more than one surgeon and/or center [8,14-18].

As expected from other reports [8], in our patients with aortic stenosis, the cross-clamp, cardiopulmonary bypass and operative times were respectively 22, 36 and 55 minutes longer for isolated full-root aortic replacement by stentless aortic xenografts compared to isolated standard stented pericardial aortic valve implantation. Nevertheless, early morbidity and mortality were not adversely affected by the prolongation of the ischemic, cardiopulmonary bypass and operative times in the full-root stentless group. In particular, the incidence of re-exploration for bleeding, complete heart block requiring definitive pace maker implantation, stroke, deep sternal infection or early mortality were similarly very low in both groups, and compared favorably to those reported in the STS Adult Cardiac Surgery database [19]. In accordance with our experience, other authors report that full root replacement using a stentless bioprosthesis does not increase postoperative morbidity or mortality of aortic valve replacement [8,9].

In addition, our study demonstrates that by eliminating the obstructive element of the stented pericardial aortic valves due to the sewing ring, the full-root implantation of stentless aortic xenografts potentially allows the insertion of an aortic valve 3–4 mm larger in diameter, i.e. two sizes larger for a given patient. However, this advantage of the stentless over stented pericardial valves disappears with the sub-coronary implantation of the former ones. Indeed some authors report no hemodynamic advantage of the stentless over stented valves [16-18]. Our results support the hypothesis that the sub-coronary implantation of the stentless valves in these studies may largely explain this finding. In our patients with aortic stenosis, full-root stentless aortic xenograft implantation provided a significantly higher IEOA than if they had received a stented pericardial valve 3–4 mm smaller. Conversely, in our patients with aortic stenosis, implantation of stented pericardial valves yielded significantly lower IEAO than if they had received a full-root stentless aortic valve 3–4 mm larger. Indeed, at one-year follow-up, full-root stentless valves of any size had significantly lower transvalvular gradients than their stented pericardial counterparts. This finding accounts for the significantly better regression of the left ventricular mass index in our patients who received full-root stentless valves as compared to those who underwent aortic valve replacement with stented pericardial valves. This finding issued from a retrospective study is in agreement with and supports the results of the prospective observational study of Fries and co-workers reporting lower transvalvular gradients at rest and during exercise as well as better regression of the left ventricular mass index in patients undergoing full-root 23-mm stentless versus 23-mm stented aortic valves [20].

### Limitations of the study

There are several factors that may influence the regression of LVH after AVR. However, the effect of many of these factors on the regression of LVH after AVR remains controversial. Whereas some authors reported no relation between the age of the patient at the time of surgery and the regression of LVH after AVR [21-24], Lund and co-workers found that older age (included in a pre-operative risk model) was indirectly associated with incomplete regression of LVH after AVR [25].

The impact of the gender is also debated. Kühl and coworkers reported that the regression of LVH after AVR was not related to gender [23] whilst Gelsomino and co-workers found that the male gender negatively affected the regression of LVH after AVR [26]. In the latter study, age did not affect the regression of LVH after AVR [26]. In our study despite the larger number of male patients in the stentless compared to the stented group, the regression of LVH after AVR was better.

Further, on one hand Tasca et al. reported a higher pre-operative LVMI to be predictor of a greater regression of LVH after AVR [24]. On the other hand Lund et al. studied transmural biopsies taken during AVR and found that the regression of LVMI was inversely related to ultrastructural changes of advanced myocardial disease represented by muscle cell diameter, nucleus volume, percent fibrosis, muscle cell and fibrous tissue mass index at the time of AVR. They concluded that the extent of LVMI regression seemed to be related to the presence of irreversible myocardial damage [27]. In this regard, patients with higher pre-operative LVMI are not likely to have less irreversible myocardial damage than those with lower pre-operative LVMI. This finding of Lund and co-workers does not support the notion that patients with higher pre-operative LVMI would necessarily show a greater regression of LVH after AVR.

In the present study, we elected to use the projected IEOA rather than the postoperative IEAO to predict PPM for several reasons. The IEOA measured by Doppler echocardiography after operation may be influenced by several factors (including subvalvular geometry, the orientation of the prosthesis, the nonuniformity of the subvalvular velocity profile, and measurement errors) [28]. In contrast, the projected IEOA is not affected by these factors and is not operator dependent. And more importantly, it can be calculated at the time of operation to predict PPM and can thus be used to prevent PPM as shown in previous studies [29,30]. In this regard, many studies that have analyzed the impact of PPM on postoperative outcomes have used the projected IEOA to define PPM. These reports [24,31,32] have further demonstrated that the projected IEOA correlates well with postoperative resting and exercise transprosthetic gradients and that it can thus be used to identify the patients who might have a high gradient on the basis of PPM.

## Conclusion

In conclusion, despite the limitations lying on the retrospective character of this study, we may submit that full-root stentless aortic valve replacement with xenografts can be performed without adversely affecting the early morbidity or mortality in patients operated on for aortic valve stenosis provided that the coronary ostia are not heavily calcified. The additional time necessary for the full-root implantation of the stentless valve compared to the implantation of the pericardial stented valves is therefore not detrimental to the early clinical outcomes and is largely rewarded in patients with aortic stenosis by lower transvalvular gradients at mid-term and a better regression of their left ventricular mass index.

## Abbreviations

IEOA: Indexed effective orifice area
LVMI: Left ventricular mass index
PPM: Patient-prosthesis mismatch
STS: Society of thoracic surgeons
LVH: Left ventricular hypertrophy
AVR: Aortic valve replacement
